# Levator anguli oris muscle based flaps for nasal reconstruction following resection of nasal skin tumours

**DOI:** 10.1186/1477-7819-9-23

**Published:** 2011-02-18

**Authors:** Adel Denewer, Omar Farouk, Tamer Fady, Fayez Shahatto

**Affiliations:** 1 Surgical Oncology Department, Oncology Center, Mansoura University, Egypt

## Abstract

**Background:**

surgical excision remains the best tool for management of skin tumors affecting nasal skin, however many surgical techniques have been used for reconstruction of the nasal defects caused by excisional surgery. The aim of this work is the evaluation of the feasibility and outcome of levator anguli oris muscle based flaps.

**Methods:**

Ninety patients of malignant nasal skin tumours were included in this study. Age was ranged from four to 78 years. For small unilateral defects affecting only one side ala nasi, levator anguli oris myocautaneous (LAOMC) flap was used in 45 patients. For unilateral compound loss of skin and mucus membrane, levator anguli oris myocautaneous mucosal (LAOMCM) flap was used in 23 patients. Very large defects; bilateral either LAOMC or LAOMCM flaps combined with forehead glabellar flaps were used to reconstruct the defect in 22 patients.

**Results:**

Wound dehiscence was the commonest complication. Minor complications, in the form of haematoma and minor flap loss were managed conservatively. Partial flap loss was encountered in 6 patients with relatively larger tumours or diabetic co-morbidity, three of whom were required operative re-intervention in the form of debridement and flap refashioning, while total flap loss was not occurred at all.

**Conclusions:**

Immediate nasal reconstruction for nasal skin and mucosal tumours with levator anguli oris muscle based flaps (LAOMC, LAOMCM) is feasible and spares the patient the psychic trauma due to organ loss.

## Introduction

The skin is the most common site of cancer development in humans. More than one million new skin cancer cases are diagnosed in the United States annually, compared with about 1.3 million cases of all other types of cancer combined. Therefore, skin cancers constituted fully one-half of all cancers diagnosed [1].

The nose, being exposed to sun light, is a common site for skin malignancy. Surgical excision remains the best tool for management of skin tumors affecting nasal skin, reconstruction of defects caused by excisional surgery have been done using many techniques including median and paramedian forehead flaps [2], Rhombic bilobed flap, and other advancement flaps [3].

The modern era of nasal reconstruction has brought significant advancements and offers unparalleled opportunities for reconstructive surgeons to maximize functional and aesthetic outcomes [4]. The forehead flap has been used for many centuries and remains a workhorse flap for major nasal resurfacing [4].

The scalping forehead flap, with the aim of using it in total nasal reconstruction, has a rich net of arterial and venous vessels that constitute the basic pattern of its blood supply through three principal pedicles: superficial temporal, supraorbital, and supratrochlear. It was described for nasal reconstruction, but due to its characteristics, such as colour of the frontal skin, texture, hairless skin, and reliable perfusion, it can be used in the reconstruction of other facial areas [5]

Burget is correctly credited with bringing the science of major nasal reconstruction to a new level. He developed a method of nasal reconstruction emphasizing the use of thin but highly vascular local lining and cover flaps to allow successful primary placement of delicate cartilage grafts. The cartilage fabrication provides projection in space, airway patency, and, when visible through conforming skin cover, the delicate contour of the normal nose. Because tissue is replaced in kind and quantity, the need for multiple revisions to sculpt and debulk is decreased [6].

When performing aesthetic restoration of the nose, the reconstructive surgeon must take into account the concept of nasal aesthetic subunits. The nose is made of alternating concave and convex surfaces, or subunits, which are separated from one another by depressions and elevations of the surrounding nasal skin. When a large portion of a given subunit has been lost, replacing the entire subunit rather than simply patching the defect often produces a superior aesthetic result. This approach places the scars of flaps and grafts within the normal depressions and elevations of the nose where they are best camouflaged.

**Figure 1:**
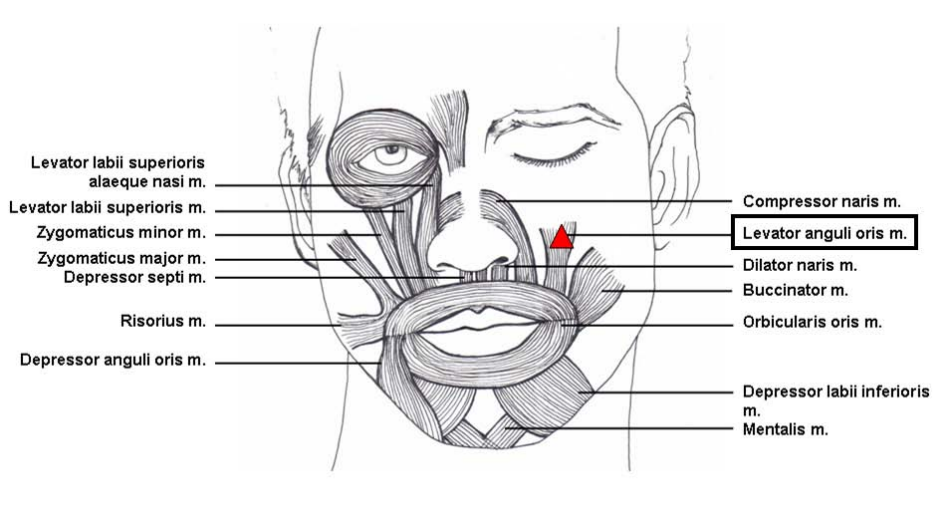
**Levator anguli oris muscle among other facial muscles**.

Levator anguli oris muscle raises the angle of the mouth and assists in producing the nasolabial furrow; it arises from the canine fossa, just below the infraorbital fossa, and is inserted in the angle of the mouth (Figure 1), intermingling with the fibers of the zygomaticus major, depressor anguli oris, and orbicularis oris.

Levator anguli oris muscle based flaps are new flaps that we are the first authors who defined it. The aim of this work is to evaluate the feasibility and outcome of levator anguli oris muscle based flaps (LAOMC, LAOMCM), in combination with other flaps when needed, in the nasal reconstruction after excision of malignant tumors.

## Patients and Methods

Over the period between July 2007 and July 2010, ninety patients of malignant skin tumours located in the nasal skin were enrolled in this study at surgical oncology unit, Mansoura University. They included 63 patients with primary lesions and 27 with recurrent tumors. There were 51 males and 39 females. Age of the patients was ranged from four to 78 years. Young aged patient were those having Xeroderma pigmentosa (9 patients). BCC was presented in 56 patients, while squamous cell carcinoma presented in 33 patients and one patient had melanoma (Table 1). Patients with advanced age or extensive comorbidity were excluded from this study.

**Table 1:** Patients characteristics:

	Number (90)	%
Male	51	56.6
Female	39	43.4

Tumour pathology:		
B.C.C.	56	62.2
Sq.C.C	33	36.7
Melanoma	1	1.1

Patients with XDP	9	10

Primary tumour	63	70
Recurrent tumours	27	30

Wide local excision with three dimensional safety margins was carried out and was guided by intraoperative frozen section in all patients prior starting the reconstructive procedure. Any infiltrated margin was dealt immediately by re-excision.

### Nasal Reconstructive Technique

**Figure 2:**
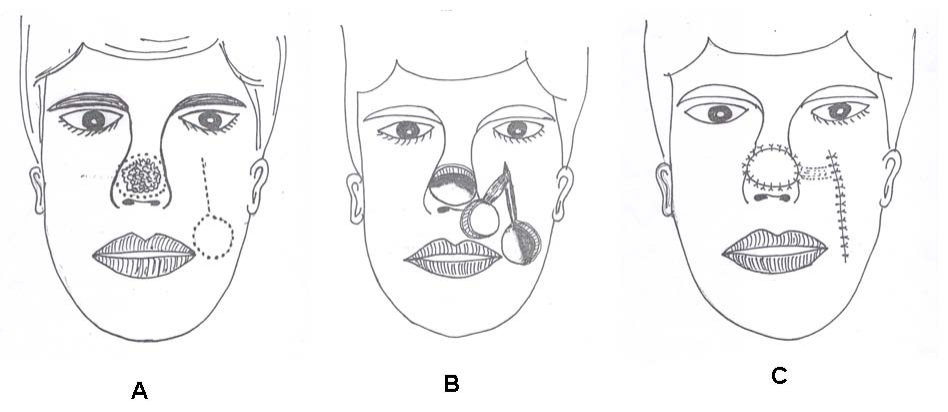
**Illustration of the operative technique of levator anguli oris muscle based flaps:** 2a: design and planning. 2b: surgical elevation of the flap. 2c: closure of defects.

**Figure 3:**
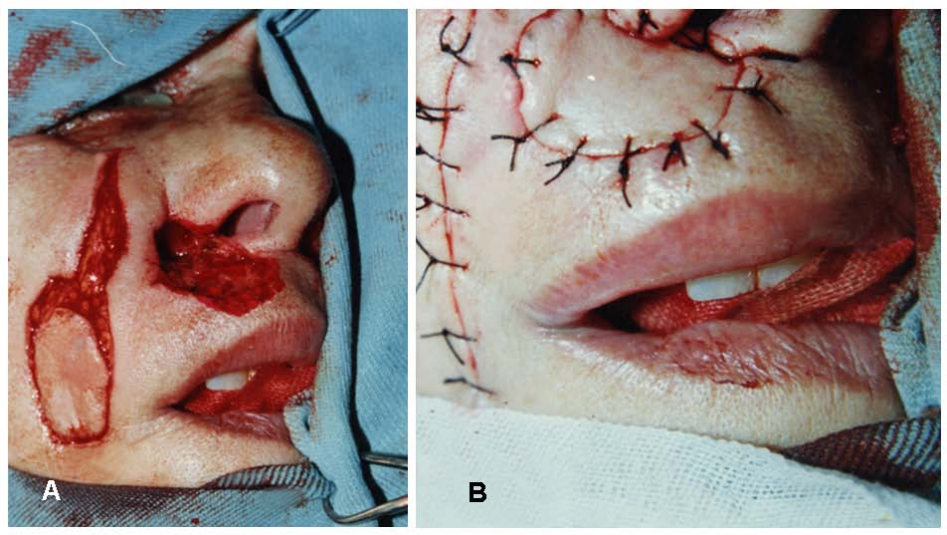
**Operative illustration of the LAOMC flap**. 3a: design and planning. 3b: closure of defects.

A skin paddle countered to the size and shape of the nasal defect was outlined in the nasolabial fold (Figure 2a &3a), it was positioned along the nasolabial fold to permit the transposition of the flap to reconstruct the nasal defect without tension. The defect location and the infraorbital rim determined its position; it is about 1 cm below the orbital rim, which is the pivot point of the pedicle.

An incision is made through the skin around the borders of the outlined skin paddle, and then from uppermost border of the skin paddle another incision was made upwards for 3-5 cm (Figure 2b). Skin flaps were raised widely in the subdermal fat to get the levator anguli oris myocautaneous (LAOMC) flap.

When the mucous membrane was desired for compound nasal loss of skin and mucous membrane, the incision around the skin paddle was deepened to oral mucous membrane with a piece of gauze inside the mouth cavity till a part of mucous membrane equal to that of the defect was included in the levator anguli oris myocautaneous mucosal (LAOMCM) flap.

The dissection was continued upwards below the levator anguli oris taking care not to injure the infraorbital artery which lies between the levator anguli oris above and the levator labii superioris below, as it emerges from the infraorbital foramen, so the dissection continued to 1 cm below the orbital margin where the vascular pedicle can be seen and preserved.

The skin bridge between the flap and the defect was elevated to create a subcutaneous tunnel and deliver the skin paddle into the defect through it or was pedicled above the skin, and later on transected after 10 to 15 days.

The mucous membrane of the levator anguli oris myocautaneous mucosal (LAOMCM) flap was first sutured to the mucous membrane of the nose and then the skin of the flap was sutured to the nasal skin (Figure 2c &3b).

The defect of the donor site is hidden in the nasolabial fold that is closed easily and primarily using polyglactin 3/0 and then subcuticular closure of the skin, but when a part of the mucus membrane of the oral cavity mucosa is also being transferred, it is closed first.

**Figure 4:**
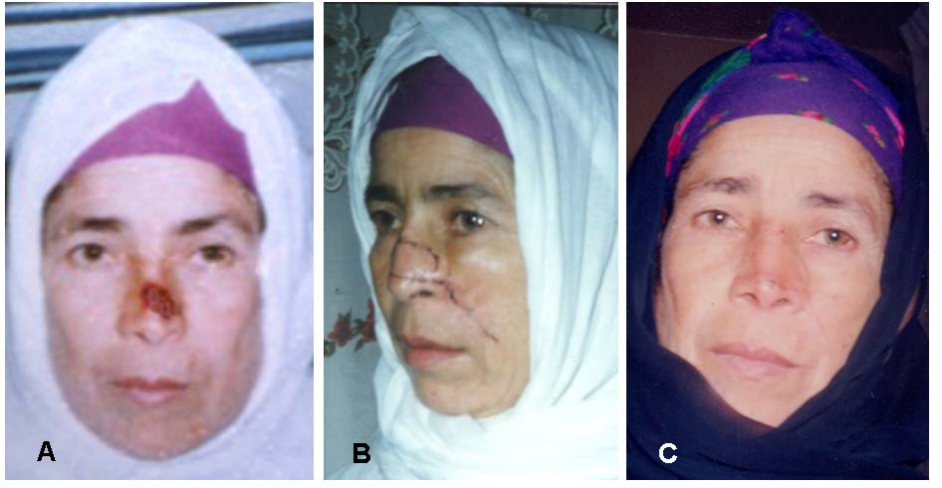
**A 47 years woman presented with BCC on the left side of the nasal skin, to whom a pedicled LAOMC flap was used to reconstruct the nasal defect**. 4a: preoperative. 4b: two weeks postoperative. 4c: two months postoperative.

**Figure 5:**
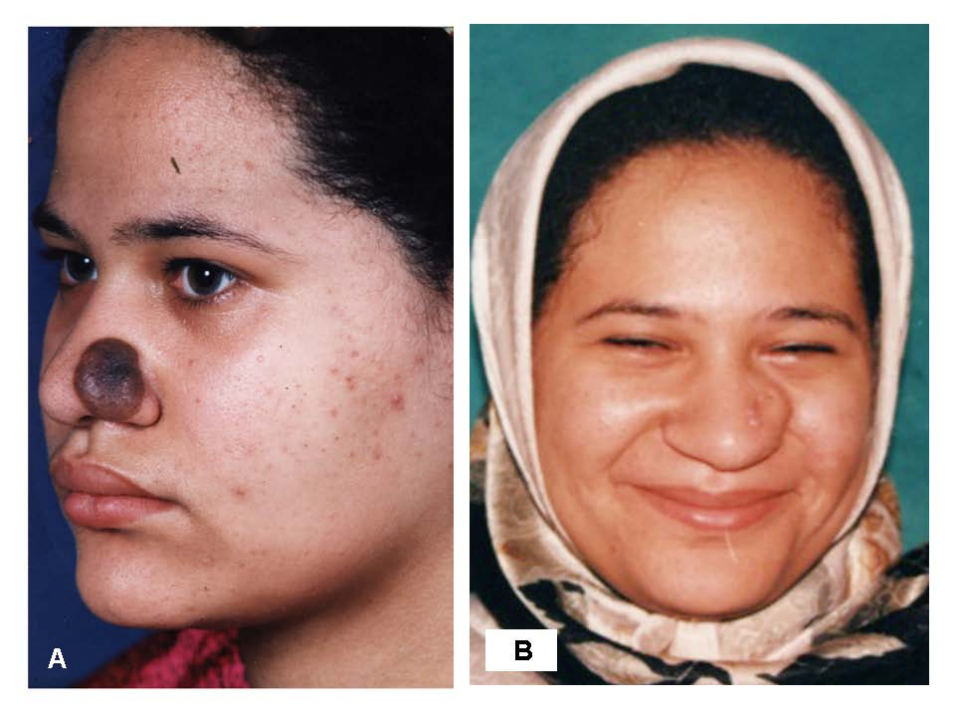
**A 26 years old female presented with melanoma to whom a tunneled LAOMC flap was used to reconstruct the nasal defect**. 5a: preoperative. 5b: one month postoperative.

**Figure 6:**
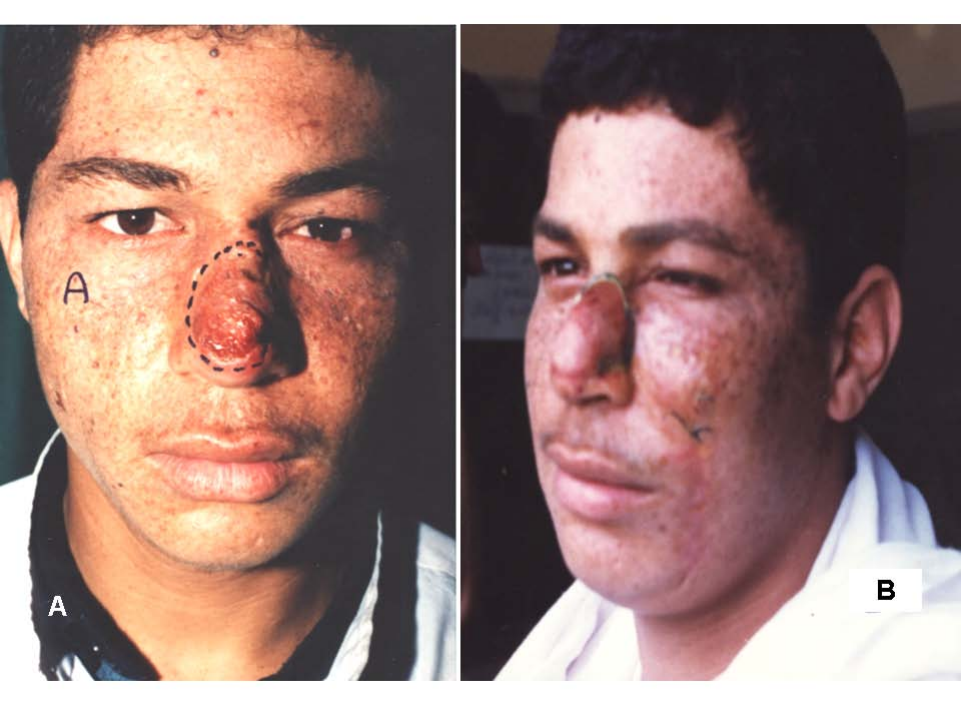
**A 17 years XDP boy presented with BCC at the dorsum of nasal skin treated with a tunneled LAOMC flap**. 6a: preoperative. 6b: three weeks postoperative.

Types of nasal reconstruction were as follows:

1- For small unilateral defects affecting only one side ala nasi, levator anguli oris myocautaneous (LAOMC) flap that includes both skin and muscle was used in 45 patients (Figure 4), of them 16 patients with tunneled flap (Figure 5 &6).

**Figure 7:**
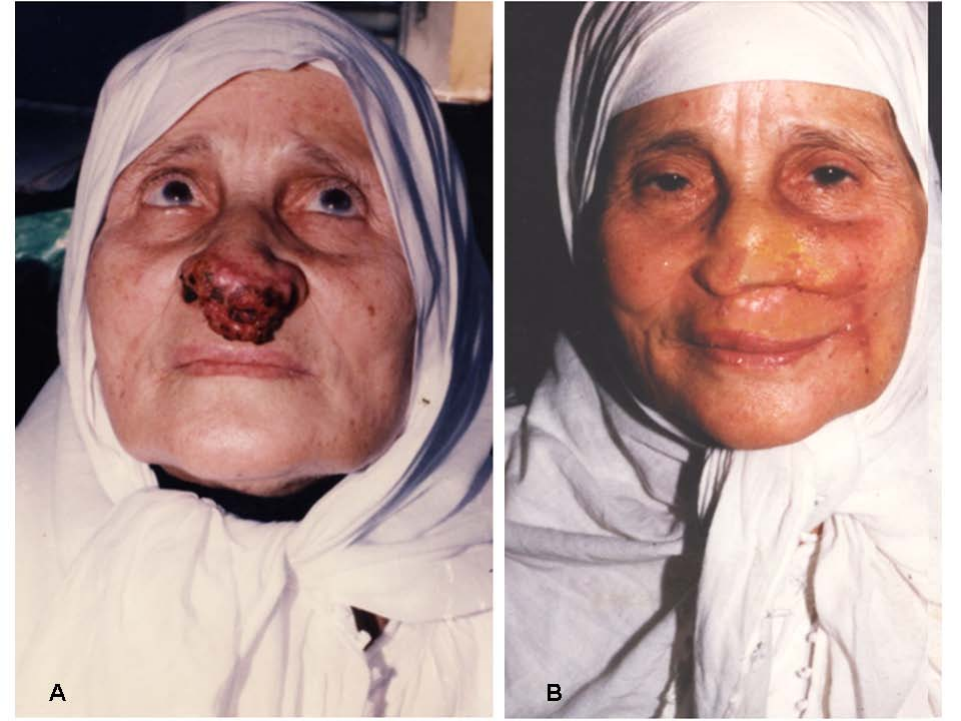
**A 62 years woman presented with BCC at tip of the nose encroaching on the left side with unilateral compound loss of skin and mucus membrane and treated with a pedicled LAOMCM flap**. 7a: preoperative. 7b: six weeks postoperative.

2- For unilateral compound loss of skin and mucus membrane, levator anguli oris myocautaneous mucosal (LAOMCM) flap (Figure 7) ± other advancement flaps depending on the site and size of the defect, was used in 23 patients.

**Figure 8:**
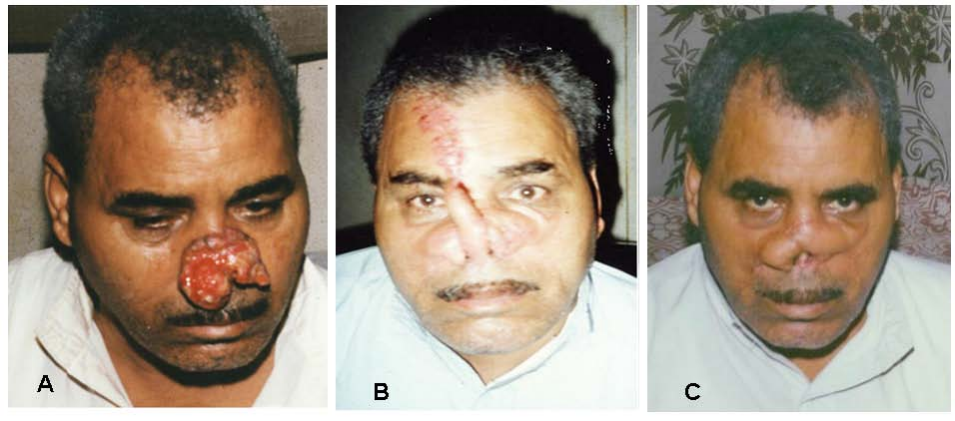
**A 50 years man presented with large advanced tumor with bilateral compound loss of skin and mucus membrane that treated with combined bilateral LAOMCM flap with supraorbital glabellar flap**. 8a: preoperative. 8b: three weeks postoperative. 8c: three months postoperative.

3- Very large defects; bilateral either LAOMC or LAOMCM flaps combined with forehead glabellar flaps (Figure 8) were used to reconstruct the defect in 22 patients.

Routine immediate and late follow up was undertaken for evaluation of the viability of the cover method, the degree of success of coverage, recipient and donor sites morbidity, operative time, hospital stay, immediate and late overall morbidity and mortality, and finally tumour recurrence within the follow up period.

## Results

Ninety patients with pathologically proven malignant nasal skin tumours were enrolled in this study. Patients' age ranged from four to 78 years (median, 40.5). Pathologic types were: 56 patients of BCC, 33 patients of squamous cell carcinoma and one patient had melanoma (Table 1). Average operating time was 1.5 - 2.5 hours and the average hospital stay was 6-8 days.

Complications are summarized in (Table 2). Wound dehiscence was the commonest complication, it accounts for 7.7% of all complications and only 2 out of 7 patients were liable to wound re-suturing. Minor complications, in the form of haematoma (3 patients) and minor flap loss (5 patients) were managed conservatively. Partial flap loss was encountered in 6 patients with relatively larger tumours or diabetic co-morbidity, three of whom were required operative re-intervention in the form of debridement and flap refashioning, while total flap loss was not occurred at all.

**Table 2:** The complications:

Complications	Number	%
Partial flap loss	6	6.6
haematoma	3	3.3
wound dehiscence	7	7.7
Unilateral nasal narrowing	2	2.2
Minor flap loss	5	5.5
Wound sepsis	4	4.4

Subjective patient satisfaction was excellent in 50, good in 28, fair in ten and poor in two cases. Patients were followed for a median of 22.4 (range; 6-36) months. During this period, no episode of local recurrence was observed.

## Discussion

The nose is not only the centrepiece of focus of the face for aesthetic reasons, but it is also critical in maintaining an adequate airway for breathing.

Advanced nasal skin tumours are not uncommon and can be cured with aggressive wide excision [7]. Intraoperative frozen section evaluation of safety margins is important before starting reconstruction to ensure complete tumour resection and decrease local recurrence rate.

The position of the nose as the focal point of the face makes its reconstruction a procedure requiring acute attention to detail and to preservation of the nasal three-dimensional integrity. Reconstructive procedures on the nose range from a straight forward direct linear closure to a complex multistage procedure requiring reconstruction of the internal lining and the cartilage support of the nose, as well as the external covering.

The scalping flap thus has several advantages over other options for nasal reconstruction. For all but the largest defects, skin for the permanent defect can be taken from the upper and lateral portion of the forehead, thus minimizing the visible scar. The donor defect can be covered with a full thickness skin graft from the retroauricular or supraclavicular region, which gives a good colour and texture match. Most of the incision is behind the hairline, and once the pedicle of the flap is divided at the second stage, the hair-bearing scalp skin is returned, leaving scars, however it needs at least two-stage procedure but the final result can be acceptable. It can be used when other flaps are contraindicated and in case of advanced lesions either alone or combining it with other techniques [8].

Forehead flap either median or paramedian provides ample skin, which matches the missing skin in both texture and thickness, it is relatively simple in concept. However, we found the only disadvantage is that it needs at least two stage procedure and sometimes require a touch up surgery to provide the possible cosmetic outcome [9], this flap provides adequate tissue bulk as the there is no need to replace missing cartilage, this was also found by Burget et al 1994 [10].

As the need to replace a whole missing aesthetic nasal unit, this was dependant on patient type and the type of the defect as well, in general especially in patients with Xeroderma pigmentosa only limited surgery provides better surgery which also may be applied for some other patients [9].

Naoshige described a method to repair full thickness defects of the nose using a glabellar flap as the lining of the nasal cavity and an expanded forehead flap for external closure. He considered his method useful in the reconstruction of a nose with a full thickness defect for which the flap donor site is limited. In our series, Glabellar flap gave good aesthetic results and it has a large available donor area that makes its use very important in case of large defects resulted from excision of locally advanced tumors [11].

The nasal lining and cartilage support is another issue of challenge in the field of nasal reconstruction. Cartilage grafts of septal, auricular or costal origins could be used, which are easy to shape and resistant both to infection, and to resorption. Moreover, the auricular cartilage is a source of grafts for reconstruction of all the cartilaginous structures of the nasal pyramid [12-14].

When the alar defect is large, the composite free flap from the root of the helix provides cover, framework and lining reconstruction of the ala and the columella as well [15-18].

The aim of cartilage grafts is to be shaped in order to emulate the external form of each subunit and to prevent sidewall collapse as well as soft tissue retraction. In many cases in our study, we preserve a part of central nasal cartilage, which is considered as a natural barrier, and remove only the infiltrated parts. However, in some cases, we could not preserve this cartilage and subsequently this affects their cosmetic result outcome for somewhat.

In case of small superficial lesions, we preferred the use of either full thickness skin grafts or other small local flaps. In more complex situations, the use of more than one flap is used as we can combine the use of forehead flap with cheek advancement flaps in reconstructing a defect resulted from excision of a lesion in the nasal ala that extends to the cheek by this combination. The angle between the nose and cheek is preserved and the cosmetic outcome is much better.

The use of LAOMCM flap can combine the reconstruction of the nose from both the mucosal surface and nasal skin in only one flap, which minimizes the risk of suboptimal reconstruction and makes the reconstruction much easier. It gives the best cosmetic result in case of small lesions, which require full thickness reconstruction. Another advantage of this technique that it can be easily used either unilaterally or bilaterally for larger and central defects. It gives very acceptable donor site scar result.

The only disadvantage noted with the use of LAOMCM flap is the loss of the angle between the cheek and the nose which is straightened in contradiction to forehead flaps which can preserve this angle.

Depending upon the patient's anxieties and self-image, the nose can be the most difficult area of the face to repair to a patient's satisfaction. Often the most difficult cases are those involving patients with small defects of the nasal tip. These patients expect little or no scar to result from their reconstructive surgery. Defects as small as 4-5 mm may represent the reconstructive surgeon's greatest challenge in terms of meeting patient expectations. Neither the degree of surgery required in a forehead flap nor the skin mismatch that can often result from grafting techniques is easily understood by the patient with a relatively small lesion.

## Conclusion

Nasal reconstruction at the time of surgery for nasal skin tumors is feasible by using levator anguli oris muscle based flaps (LAOMC, LAOMCM), and spares the patient the psychic trauma due to organ loss; it is oncologically safe after frozen section examination of the resected tumor.

## Competing interests

The authors declare that they have no competing interests.

## Authors' contributions

AD carried out the surgical techniques, conceived of the study and drafted the manuscript. OF participated in the design of the study, drafted the manuscript and assisted in surgical techniques. TF participated in the design of the study, drafted the manuscript and assisted in surgical technique. FS performed the statistical analysis, and participated in its coordination. All authors read and approved the final manuscript.
